# Long-pulsed ultrasound-mediated microbubble thrombolysis in a rat model of microvascular obstruction

**DOI:** 10.1515/med-2024-0935

**Published:** 2024-03-27

**Authors:** Rui Wang, Xianghui Chen, Daogang Zha

**Affiliations:** Department of Cardiology, Nanfang Hospital, Southern Medical University, Guangzhou, China; Department of Cardiology, The First Affiliated Hospital of Jinan University, No. 613 Huangpu West Avenue, Guangzhou, Guangdong, China; Department of General Practice, Nanfang Hospital, Southern Medical University, Guangzhou, China; Department of Ultrasound, Guangdong Provincial Hospital of Traditional Chinese Medicine, Guangzhou, China

**Keywords:** thrombolysis, microbubble, microvascular obstruction, long-pulsed ultrasound

## Abstract

In up to 30% patients who experience acute myocardial infarction, successful recanalization of the epicardial coronary artery cannot provide adequate microvascular reperfusion. In this study, we sought to determine whether long-pulsed ultrasound (US)-mediated microbubble (MB) cavitation was useful for the treatment of microvascular obstruction, and the therapeutic effects were compared within different long-pulse-length and short-pulsed US. Microvascular obstruction model was established by injecting micro-thrombi into common iliac artery of a rat’s hind limb. About 1 MHz US with different long pulse lengths (ranging from 100 to 50,000 cycles) was delivered, compared to short pulse (5 cycles). The control group was given MB only without therapeutic US. Contrast perfusion images were performed at baseline, emboli, and 1, 5, 10 min post-embolization, and peak plateau video intensity (*A*) was obtained to evaluate the therapeutic effects. Long-tone-burst US showed better thrombolytic effects than short-pulsed US (1,000, 5,000 cycles >500 cycles, >5 cycles, and control) (*P* < 0.01). 1,000 cycles group showed the optimal thrombolytic effect, but microvascular hemorrhage was observed in 50,000 cycles group. In conclusion, long-tone-burst US-enhanced MB therapy mediated successful thrombolysis and may offer a powerful approach for the treatment for microvascular obstruction within a certain pulse length.

## 1 Introduction

Previous studies have shown that successful recanalization of the epicardial coronary artery by percutaneous coronary intervention (PCI) does not achieve adequate microvascular reperfusion in up to 30% patients with acute ST-segment elevation myocardial infraction (STEMI), a phenomenon known as “no-reflow” [1–3]. No-reflow is a complex multifactorial phenomenon, potential treatable components include microvascular obstruction, platelet aggregation, microcirculatory spasm, etc. Patients with no-reflow are associated with higher incidence of malignant arrythmia, refractory heart failure, progressive left ventricular remodeling, and sudden death [4].

Ultrasound (US)-assisted microbubble (MB) therapy has been investigated as a therapeutic approach for the treatment of occlusive thrombi in acute myocardial infarction [5]. The US-enhanced MB therapeutic effects have been found to facilitate thrombolysis under different experimental conditions: with recombinant tissue plasminogen activator (rt-PA) [6] and without rt-PA [7,8], with 40–500 kHz low frequency [9] or 1–5 MHz high frequency [10]. Presently, the US used in clinic is usually short cycle (≤5 µs). A recent research showed a 20 µs long-pulsed duration US with MB infusion was able to recanalize acutely thrombosed carotid arteries and restore downstream flow [11]. Different from short-pulsed US, 30 µs pulse-length US was used to augment limb perfusion in patients with peripheral artery disease [12]. The ultra-high-speed imaging system further confirmed large-amplitude MB oscillations under inertial cavitation caused thrombus deformation and pitting [13]. In an *in vivo* study, 5,000 µs long-tone-burst US-assisted MB therapy was proved to be an important strategy for the treatment of microvascular micro-embolization [14]. Our study showed long-pulse-length US with MB became a valuable therapy for reperfusion of microvascular obstruction, and the persistent cavitation activity of the MBs during long-tone burst US was an important mechanism and conferred excellent reperfusion efficacy [15]. Compared to short-pulse US, longer pulse drove greater MB cavitation and more rapid microvascular flow rate restoration after thrombotic obstruction [16]. Nitric oxide played a significant role in the MB oscillation-induced sonoreperfusion efficacy [17]. The MB acoustic behaviors caused sustained enhancement of cellular and vascular permeability through a physical, cavitation-based mechanism of sonoporation [18].

In this study, a microvascular obstruction model on a rat hind limb was made as previously described [14]. Long-pulsed US-mediated MB cavitation was used for the treatment of microvascular obstruction, and the therapeutic effects were compared with traditional short-pulsed US.

## 2 Methods

### 2.1 Preparation of micro-thrombi

Fresh rat blood was mixed with coagulation accelerator (1:10) from lyophilizing thrombin powder (Thrombin-1000U, Biosharp, Guangzhou, China) and 5% CaCl_2_ solution. Then the blood was placed in a glass vial and incubated at room temperature for 24 h until fully coagulated. The clots were then passed through mesh with small pores (TS-BXGSW, Filter Manufacture, Shanghai, China) to make micro-thrombi of about 30–100 μm size as in our previous study [19].

### 2.2 Microvascular obstruction model

All experimental procedures in this study were granted by ethics board of Southern Medical University (Guangzhou, China) in compliance with the institutional guidelines for the care and use of animals. Sprague Dawley (SD) male rats (*n* = 42) weighing 280 ± 22 g (age of 8–9 weeks) were anesthetized with isoflurane (3%) and placed on a thermostat plate to keep the temperature constant at 37℃. The right external jugular vein was cannulated with a polyethylene 50 tubing for infusion of lipid-perfluoropropane MBs. Mixture of dipalmitophosphatidylcholine, dipalmitophosphaacid, dipalmitoylphosphatidy-lethanolamine-polyethylene glycol5000 were sonicated in the presence of perfluorobutane gas to produce MBs. The Coulter counter showed the MB was with a mean diameter of ∼2.5 μm and a concentration of ∼3 × 10^9^/mL. Another PE-50 tubing was placed in the left common iliac artery, where micro-thrombi suspension (0.5–0.75 ml) was injected to induce micro-embolization in the right hind limb. The right hind limb was deployed for better visualization and US delivering. Successful micro-embolization model was established by visual observation of the bright field areas reduced by at least 50%. After 10 min, perfusion imaging was repeated to confirm persistent hind limb hypo-perfusion to avoid the spontaneous dissolution of thrombosis. Additional micro-thrombi will be injected if the bright areas recovered to >50% of baseline. Individuals who died from anesthetic accident were excluded from our study.

### 2.3 Therapeutic US

**Figure 1:**
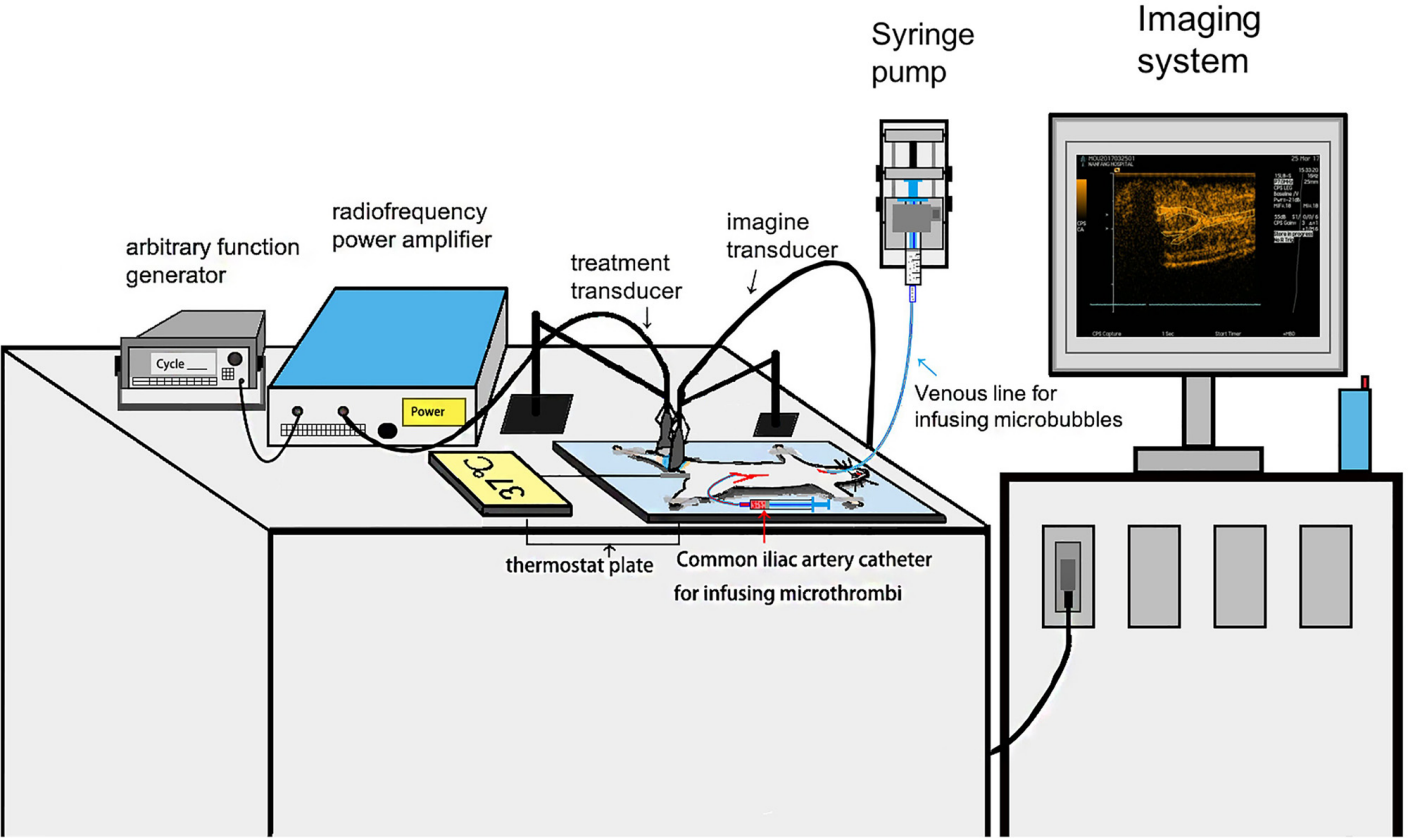
Diagram for the long-pulsed ultrasound therapeutic system (left) and ultrasound imaging system (right). The therapeutic system consists of a single-element transducer, function generator, and power amplifier. The rat was placed on a thermostat plate with right jugular vein cannulated for microbubble infusion and left common iliac artery for micro-thrombi injection.

About 1 MHz of therapeutic US (1.8 MPa, pulse interval 3 s) with different pulse-lengths was delivered from a single-element transducer (Olympus NDT, US), driven by an arbitrary function generator (33210A, Agilent Technologies, Inc., Santa Clara, CA, USA) and intensified by a power amplifier (2200L, Electronics & Innovation, Rochester, NY, USA) (Figure 1). 15L8 transducer (Acuson, Siemens, Germany) was used as imaging probe and contrast pulsed sequence mode was utilized (7.0 MHz, mechanical index 0.18). The longitudinal section of the hind limb muscle (the midpoint between groin and knee joint) was selected to obtain perfusion images at each experiment stage. Mechanical index was set at 1.9 for burst-period and at 0.18 for replenishment period. Precise alignment was confirmed by visualizing MB destruction in contrast imaging immediately after delivering therapeutic US. MBs were continuously infused at 3 mL/h and the contrast imaging of rat hind limb was analyzed offline by analysis software (MCE Version 2.7, University of Virginia, VA, USA). As in our previous study [20], different regions of interest were chosen, then video intensity and time were used to calculate *A* and *β* by function *Y*(*t*) = *A* × (1 – e^−*βt*^), where *A* stands for myocardial blood volume, *β* stands for microbubbles blood velocity, and *A* × *β* stands for microvascular blood flow [14,17].

### 2.4 Treatment protocol

A protocol was prepared before the study without registration. SD rats were randomly divided into seven groups: (1) treatment groups (*n* = 6 for each group): continuous infusion of MB and imaging ultrasound visualized, each group receiving one pulse length US (cycles number = 5, 100, 500, 1,000, 5,000 to 50,000) after embolization. (2) Control groups (*n* = 6): continuous infusion of MB and imaging ultrasound visualized, receiving no therapeutic US. For all groups, baseline perfusion imaging of hind limb was performed at the beginning of the experiment. The exposure time for therapeutic US was set at 10 min, since according to our previous experiments 10 min was enough to generate successful thrombolysis. After embolization, perfusion imaging was performed at the first, fifth, and tenth minute post-therapeutic US. Finally, rats were sacrificed and histology was performed *post mortem* in both treatment and control animals to observe long-tone-burst US-mediated thrombolysis. The skeletal muscle of right posterior hind limb was selected, and the territory transversely exposed to therapeutic US was sampled. Hematoxylin and eosin (HE) staining was used and the selected sections were examined microscopically.

### 2.5 Statistical analysis

SPSS 19.0 (IBM Corp., Armonk, NY, USA) was used for statistical analysis and data were expressed as the mean ± standard deviation. Repeated-measure analysis of variance was used to compare differences within groups and the LSD *post hoc t*-test was used for comparison of differences between groups (*P* < 0.01 was considered significant). The Bonferroni *post hoc t*-test was used to compare differences at different experiment stages, and the significance was defined at *P* < 0.002 (0.01/5) to adjust for multiple comparisons.

## 3 Result

**Figure 2:**
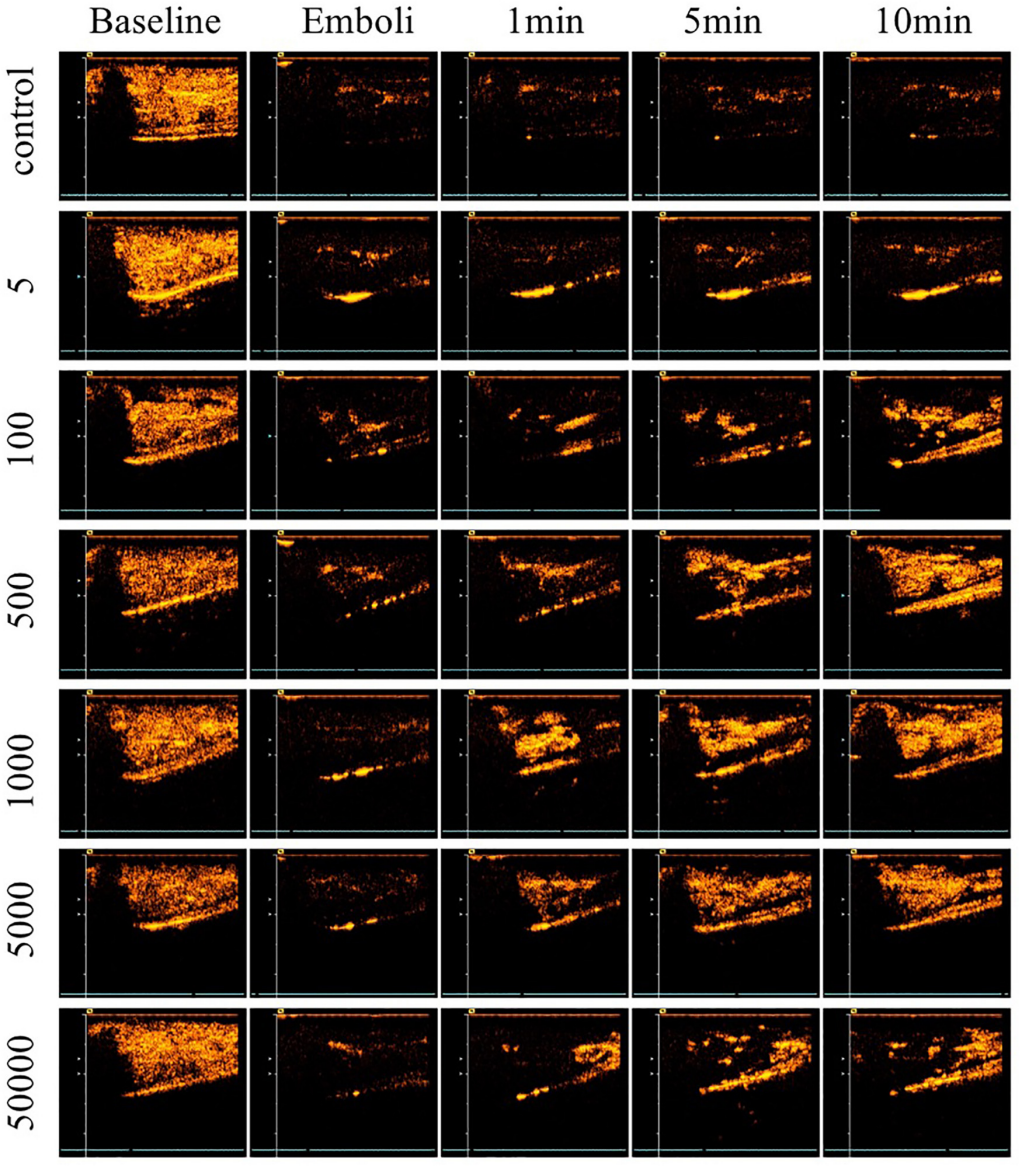
Contrast perfusion images at different experimental stages. Ultrasound pulse length increases from 5 to 50,000 cycles from top to bottom, the control group was given microbubble only with no therapeutic ultrasound. Five experimental stages are listed from left to right: baseline (before treatment), emboli (microvascular embolization), 1, 5, and 10 min post-therapeutic ultrasound.

The contrast images with peak plateau video intensity in control and all treatment groups at five experimental stages were observed: baseline (before treatment), emboli (microvascular embolization), 1, 5, and 10 min after therapeutic US or MB only (Figure 2). In the control group, a persistent decrease in perfusion was seen after micro-embolization throughout the whole experiment period. Slight improvement of reperfusion was observed in 5 and 100 cycle groups. When the pulse length increased to 500 cycles, a significant increase in video intensity was noted after delivering long-pulsed US in 500, 1,000, 5,000 cycle groups. However, when pulse length increased to 50,000 cycles, the sustainable hypo-perfusion appeared.

**Figure 3:**
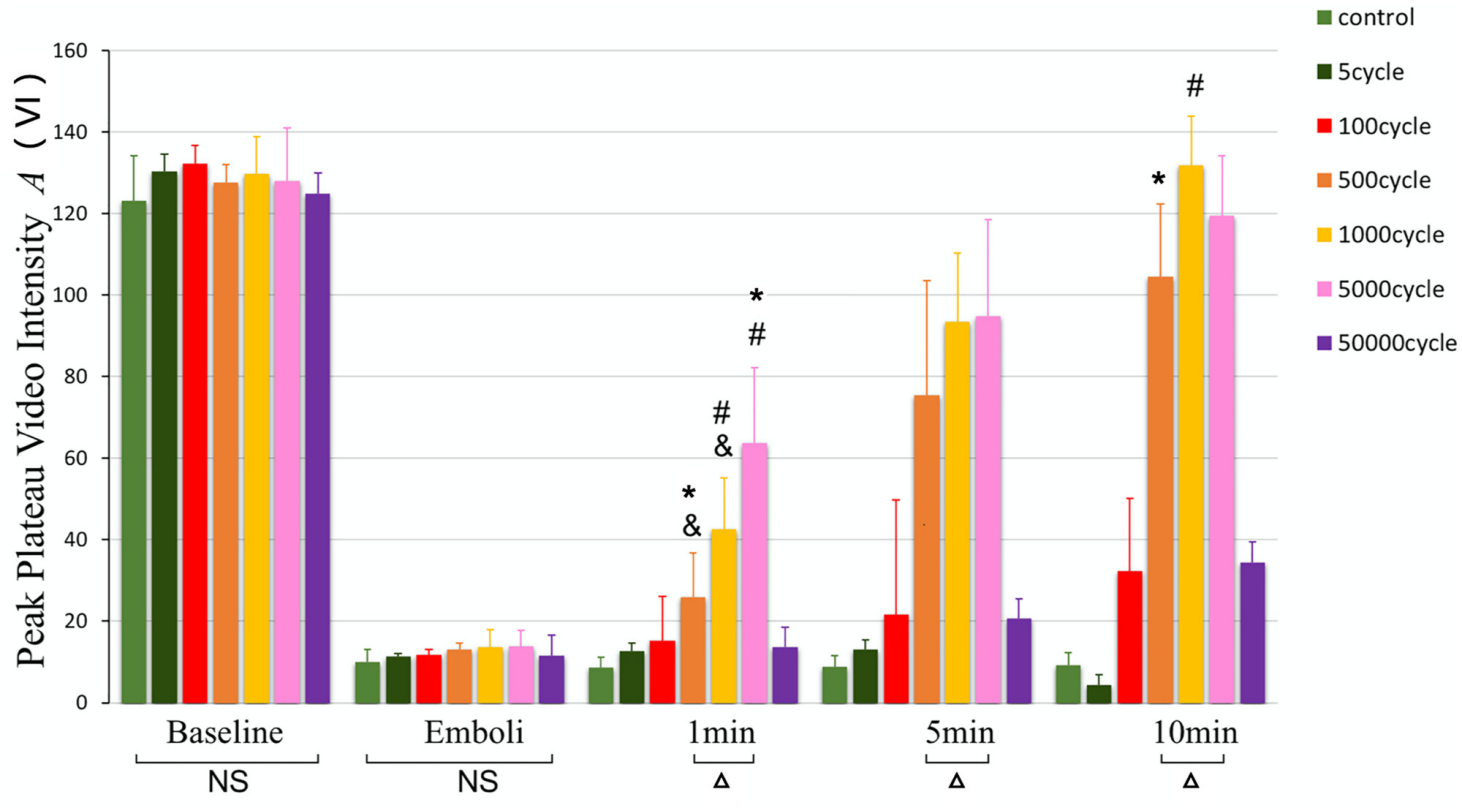
Peak plateau video intensity (*A*) at various experimental stages. There was no significant difference among groups at baseline and emboli (^NS^ *P* > 0.01). At 10 min, 500, 1,000, and 5,000 cycles were much higher than control, 5, 100, and 50,000 cycle groups (^Δ^ *P* < 0.01). ^NS^ *P* > 0.01, considered no significant differences; ^Δ^ *P* < 0.01, compared with control, 5, 100, and 50,000 cycle groups; ^#^ *P* < 0.01, compared with 500 cycles group; *P < 0.01, compared with 1,000 cycles group; ^&^ *P* < 0.01, compared with 5,000 cycles group.

Next, peak plateau video intensity among different groups was compared at five experimental stages (Figure 3). There was no significant difference among groups at baseline and emboli (NS, *P* > 0.01). At 1 min, 1,000 cycles group was higher than 500 cycles group (*P* < 0.01) but less than 5,000 cycles group (*P* < 0.01). At 5 min, 500, 1,000 and 5,000 cycle groups were significantly higher than the control group and 5, 100 and 50,000 cycle groups (*P* < 0.01). 1,000 and 5,000 cycle groups were much higher than 500 cycles group (*P* < 0.01), there was no significant difference between 1,000 cycles and 5,000 cycles group (*P* > 0.01). At 10 min, 500, 1,000 and 5,000 cycles were much higher than control, 5, and 100 cycle groups (*P* < 0.01). 1,000 cycles was much higher than 500 cycles (*P* = 0.002 < 0.01), there was no significant difference between 5,000 cycles and 500 cycles (*P* = 0.08 > 0.01), little difference between 1,000 cycles and 5,000 cycles (*P* = 0.145 > 0.01).

**Figure 4:**
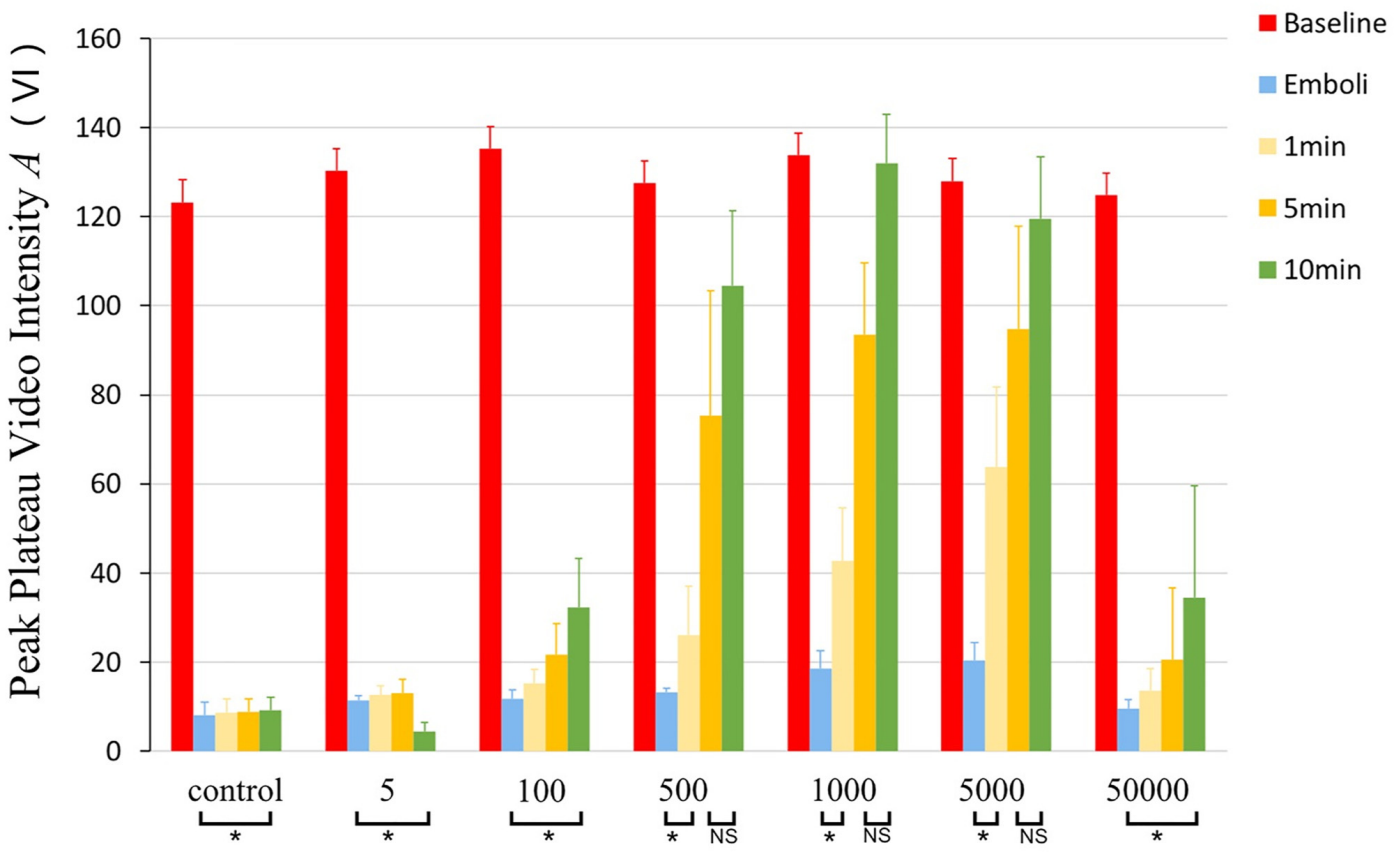
Peak plateau video intensity (*A*) in each group. In 5, 100, and 50,000 cycle groups, there was no significant reperfusion effects after therapeutic ultrasound (*P < 0.01 vs baseline). In 500, 1,000, and 5,000 cycle groups, effective thrombolysis occurred at 5 min (*P* < 0.01 vs emboli).

Moreover, the peak plateau video intensity (*A*) standing for myocardial blood volume was about 130 at baseline in all groups (Figure 4). In the control and 5, 100, 50,000 cycles treatment groups, after the micro-embolization peak plateau video intensity was no longer higher than micro-embolization and remained lower than baseline (*P* < 0.0001 vs baseline) throughout the whole experiment. In 500, 1,000 and 5,000 cycle groups, *A* became higher than embolization at 5 min and higher than baseline at 10 min (*P* > 0.01, compared to baseline).

**Figure 5:**
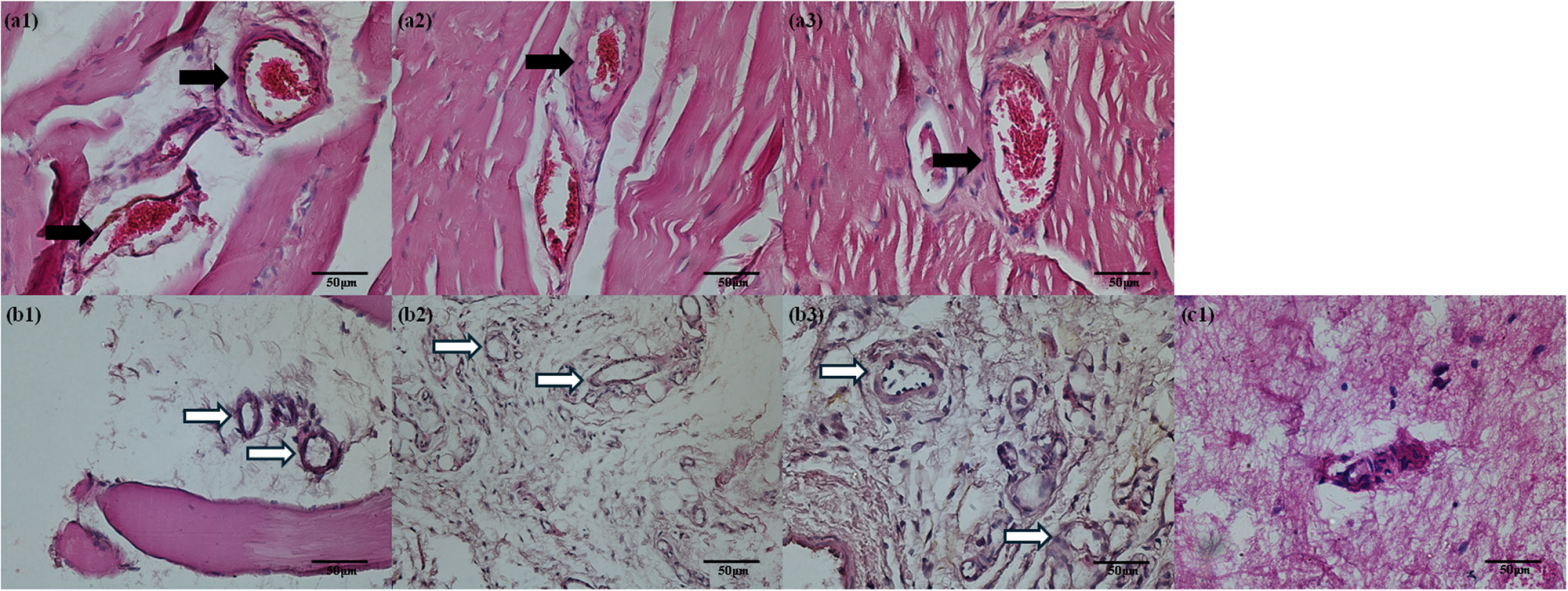
Hematoxylin and eosin-stained skeletal muscle of rat hind limb. Microvascular obstruction was seen in the control (a1), 5 cycle (a2), and 100 cycle (a3) groups, and microvessel clot lysis was found in 500 cycle (b1), 1,000 cycle (b2), and 5,000 cycle (b3) groups. Hemorrhage and architectural derangements were observed in 50,000 cycle group (c1). The black arrow indicates micro-embolization, and the white arrow indicates successful thrombolysis.

HE stained skeletal muscle of rat hind limb was performed to further confirm the therapeutic effects of this combined therapy (Figure 5). Microvascular obstruction was seen in the control (a1), 5 cycle (a2), and 100 cycle (a3) groups; meanwhile, successful thrombolysis was found in 500 cycle (b1), 1,000 cycle (b2), and 5,000 cycle (b3) groups. For the extremely long-pulsed duration 50,000 cycle group, tissue hemorrhage and architectural derangements were observed (c1).

## 4 Discussion

About 30% of patients with STEMI did not get effective reperfusion in micro-vasculatures, despite normal epicardial coronary flow by successful PCI procedures, a phenomenon named as “no-reflow” [1]. The micro-thrombi that obstruct downstream vasculature is an important reason for that, and the collapse of microvasculture induces post-infarction complications and left ventricular remodeling. Therapeutic US and intravenous MBs have been shown to dissolve micro-embolization and improve peri-infarct microvascular flow in a porcine model of STEMI, even when epicardial artery remained occluded [21]. Long-pulsed duration US with MB became a valuable adjunct to sonoreperfusion and improved the effectiveness of sonothrombolysis at low acoustic pressure [22]. It was proved in a clinical trial that the guided high mechanical index impulses with long pulse durations were able to prevent microvascular obstruction when co-administered with MBs infusion [5]. Further study revealed US with long pulse duration therapeutic impulses produced sustained cavitation events, thereby possibly causing long-lasting epicardial and microvascular re-flow in STEMI. In our study, a micro-embolization model was successfully established to mimic the no-reflow phenomenon after PCI, and long-tone-burst US showed very good capacities of dissolving micro-thrombi when augmented by lipid MBs.

To our knowledge, this is the first study to investigate the thrombolytic effect of very long-pulsed US (50,000 cycles) medicated MB cavitation *in vivo*. In short-pulse five cycles group, no recovery sign of reperfusion was observed. When the pulse length increased to 500 cycles, a numerical increase in capillary volume was seen just after giving long-pulsed US for 5 min. The 1,000 cycles and 5,000 cycle groups showed better lytic effects than 500 cycles, but there was no difference between 1,000 cycles and 5,000 cycles. Previous *in vitro* studies showed that the lytic effect increased when the pulse length was prolonged, and the best parameter was 5,000 cycles [19]. The acoustic behaviors of MBs have been well visualized by ultra-high-speed-imaging system (up to 5 million frames per second), implying acoustic radiation forces (Bjerknes forces) played a role in mediating clot disruption through inertial cavitation events [13]. And the most effective long-tone-burst US parameter in our study seemed to be a little shorter than *in vitro*. Possibly less US energy from a shorter pulse is enough to induce effective thrombolysis *in vivo*.

A thrombotic micro-embolization model on rat’s hind limb was successfully made in our study and HE staining of skeletal muscle showed obvious evidence of micro-embolization. However, there was no adequate reperfusion post micro-embolization and the histology indicated tissue bleeding when pulse length increased to 50,000 cycles. A possible explanation for this phenomenon is that long-tone-burst US causes endothelial injury when pulse length exceeds a certain limit. For US-targeted MB cavitation, it is suggested that the acoustic parameters including line density and pulse length have a maximal effect for flow augmentation [23]. A paradoxical reversal phenomenon was observed for the thrombolysis in a porcine model of acute carotid thromboembolism when the mechanical index was too high [11]. In a phase II clinical trial, long-pulsed US was found to cause coronary vasoconstriction in 50% patients after using long-pulsed duration impulses pre- and post-PCI [24]. But the probe used in our study was different from the clinical one and the pathology showed no microvascular injury or tissue bleeding under 5,000 cycles, it is necessary to detect in further studies what are the exact factors that caused long-pulsed US-induced unexpected side effects.

### 4.1 Limitations

There are several limitations in our study. First, this study did not take into account endothelial damage related to ischemia time, which can influence very varied results. Second, we focused on the thromboembolism on microvascular, but other components such as cholesterol crystals, severe atherosclerosis, and endothelial dysfunction also play important roles in no-flow phenomenon after PCI. Third, the thrombolitic markers at cellular levels such as creating kinase were not detected. Finally, the function of extremity post micro-embolization and thrombolytic treatment was not detected. The exact mechanisms of long-pulsed US-mediated thrombolysis remain not fully understood.

## 5 Conclusion

In summary, long-tone-burst US-assisted MB therapy showed successful thrombolytic effects and may offer a powerful approach for the treatment for microvascular obstruction within a certain pulse length.
